# Intraoperative ultrasound in breast-conserving surgery for palpable breast cancer: Association with surgical margins and resection volume

**DOI:** 10.17305/bb.2026.13831

**Published:** 2026-03-30

**Authors:** İsmail Zihni, Osman Cem Yilmaz, Meliha Ülkü Güzel

**Affiliations:** 1 Department of General Surgery, Akdeniz University Faculty of Medicine, Antalya, Türkiye; 2 Department of Breast Surgery, Istanbul Breast Center, Istanbul, Türkiye; 3 Department of General Surgery, Antalya City Hospital, Antalya, Türkiye

**Keywords:** Breast-conserving surgery, ultrasonography, breast neoplasms, surgical margins, neoplasm localization, intraoperative ultrasound

## Abstract

Accurate determination of surgical margins is a crucial aspect of breast-conserving surgery (BCS) for early-stage breast cancer. Traditionally, tumor localization has been performed through palpation followed by frozen section analysis. However, intraoperative ultrasonography (US) presents a practical and cost-effective alternative, particularly for small or poorly palpable lesions. This study aimed to evaluate the relationship between the use of intraoperative US and surgical outcomes in BCS. We conducted a retrospective analysis of 180 female patients with early-stage breast cancer who underwent surgery from 2020 to 2023, excluding those who received neoadjuvant therapy. Patients were classified into two groups based on the application of intraoperative US. We compared demographic, histopathological, and surgical parameters, including tumor diameter, resection volume, tumor diameter-to-resection volume ratio, closest surgical margin, and the necessity for cavity re-excision. Intraoperative US was utilized in 51.1% of cases (*n* ═ 92). Tumor size was notably smaller in the intraoperative US group compared to the palpation-guided group (median 15 mm vs. 20 mm, *P ═* 0.019). Additionally, the median resection volume was significantly lower in the US group (64.65 cm^3^ vs. 130.91 cm *P ═* 0.001). The tumor diameter-to-resection volume ratio was higher in the intraoperative US group (0.03 vs. 0.02, *P ═* 0.044), indicating more precise tumor-targeted excision. Cavity re-excision rates were comparable between the two groups (10.9% vs. 15.9%, *P ═* 0.482), and no postoperative positive margins were recorded. Intraoperative ultrasonography was associated with adequate surgical margins and reduced resection volumes in breast-conserving surgery, suggesting its potential to enhance tissue preservation without increasing re-excision rates.

## Introduction

Breast cancer is the most prevalent malignancy among women globally. Advances in medical and radiation oncology have established breast-conserving surgery (BCS) as a preferred surgical approach due to its favorable cosmetic outcomes and reduced morbidity rates [1]. A critical step in preventing local recurrence following BCS is achieving adequate resection margins, with the optimal margin width remaining a contentious issue. The 2025 National Comprehensive Cancer Network (NCCN) guidelines endorse the “no ink on tumor” principle for invasive breast cancer [2]. This principle emphasizes the complete excision of the tumor with negative margins at the specimen surface [3].

To accomplish this objective, precise preoperative assessment of tumor size and localization, along with meticulous surgical planning informed by preoperative imaging, is essential. Despite this, intraoperative determination of tumor location and size through palpation remains a commonly employed method for palpable breast lesions. Additionally, frozen section analysis for margin assessment may be selectively used, depending on institutional protocols. Inadequate surgical margins often result in secondary surgeries or intraoperative re-excision, which can prolong operative time and increase morbidity.

An alternative strategy to achieve negative margins intraoperatively is the use of intraoperative ultrasonography (US) to delineate tumor boundaries. With intraoperative US guidance, accurate identification of tumor localization, size, and margins facilitates resections that adhere to appropriate surgical margin standards. While frozen section analysis is regarded as a relatively standardized method for margin assessment during BCS, intraoperative US has not yet been widely adopted as a standard technique, primarily due to the limited number of studies evaluating its clinical effectiveness.

This study aims to investigate the association between the utilization of intraoperative US and surgical margin status, tumor stage, and the necessity for cavity re-excision in patients undergoing BCS. The present research represents an initial clinical experience focused specifically on patients with palpable breast tumors, reflecting the early implementation phase of intraoperative ultrasound in our surgical practice.

## Materials and methods

This study included 180 female patients newly diagnosed with early-stage breast cancer who underwent surgery between 2020 and 2023, did not receive neoadjuvant therapy, and had complete clinical and pathological datasets. Patients were categorized into two groups based on whether intraoperative US or palpation alone was employed during surgery. Data were collected retrospectively. All tumors included were clinically palpable lesions identified during physical examination.

Demographic evaluations of patients in both groups included age and menopausal status. Histopathological characteristics, such as T and N status, and tumor, node, metastasis (TNM) staging according to the American Joint Committee on Cancer (AJCC) 8th Edition, were collected. However, T1 subclassification (T1a, T1b, T1c) could not be consistently retrieved due to limitations in retrospective data collection. Additional histopathological variables recorded included tumor grade, lymphovascular invasion, tumor necrosis, presence of associated ductal carcinoma in situ (DCIS), estrogen receptor (ER) status, progesterone receptor (PR) status, human epidermal growth factor receptor-2 (HER2) expression, and Ki-67 proliferation index. These variables were documented to characterize tumor features and identify potential baseline differences between the study groups rather than outcomes influenced by intraoperative ultrasound. Ki-67 was routinely assessed in all patients as part of the standard immunohistochemical evaluation at our institution. Molecular subtypes were determined using surrogate immunohistochemical definitions based on ER, PR, HER2 status, and Ki-67 index, with a cut-off value of 15% applied for Ki-67 expression. ER-positive, HER2-negative tumors with Ki-67 <15% were classified as luminal A-like, while ER-positive tumors with Ki-67 ≥15% and/or HER2 positivity were classified as luminal B-like. Tumors lacking ER and PR expression and without HER2 overexpression were classified as triple-negative.

Tumor localization, size in millimeters (mm), tumor diameter, resection volume in cubic centimeters (cm^3^), tumor diameter-to-resection volume ratio, and the closest surgical margin in millimeters were documented. Tumor diameter was based on the final histopathological measurement, while resection volume was determined intraoperatively by measuring the dimensions of the excised surgical specimen. The resection volume was calculated in cubic centimeters (cm^3^). Surgical margins were evaluated by the pathology department using standard specimen orientation and inking techniques. The closest surgical margin was defined as the minimum microscopic distance between the tumor and the inked specimen margin, measured in millimeters. Margin positivity was defined as the presence of tumor cells at the inked margin for invasive carcinoma according to the “no ink on tumor” principle; for DCIS, tumor cells located at or within 2 mm of the inked margin were considered positive. Cavity re-excision referred to the additional removal of tissue from the surgical cavity during the same operation when a positive or close margin was suspected or identified intraoperatively.

Margin-related outcomes were defined using three distinct, non-overlapping concepts:

(1) Intraoperative cavity re-excision was defined as additional tissue removal from the surgical cavity during the same operation when margin adequacy was uncertain.

(2) Final pathological margin status referred to margin assessment on permanent histopathological examination based on the “no ink on tumor” principle.

(3) Secondary re-operation was defined as any subsequent surgical procedure performed following the initial surgery due to positive margins.

In this cohort, cavity re-excision was exclusively intraoperative, and no patients required secondary re-operation.

Additional parameters included axillary dissection status and the need for cavity re-excision due to positive surgical margins following the initial operation.

### Inclusion criteria

Patients were eligible for inclusion in this study if they met the following criteria:

- Female patients aged ≥18 years
- Histologically confirmed early-stage breast cancer
- Clinically palpable breast tumors detected during physical examination
- Undergoing BCS between 2020 and 2023
- No prior neoadjuvant therapy
- Availability of complete clinical and pathological datasets

Patients who received neoadjuvant chemotherapy were excluded to maintain a homogeneous study population, as neoadjuvant therapy can significantly alter tumor size and morphology, potentially impacting surgical planning, resection volume, and pathological margin assessment. Thus, only patients undergoing primary surgery without prior systemic therapy were included.

### Exclusion criteria

Patients were excluded from the study if they met any of the following criteria:

- Patients younger than 18 years or male
- Patients who received neoadjuvant therapy
- Presence of multifocal or multicentric tumors
- History of acute mastitis, breast abscess, granulomatous mastitis, or prior surgery on the same breast for benign conditions
- Patients with non-palpable lesions operated on using wire localization
- Patients with incomplete or unavailable clinical data

Patients with incomplete datasets were excluded prior to statistical analysis to ensure that all variables included in the study were available for the final cohort.

In our institution, non-palpable breast lesions are routinely localized preoperatively using wire localization under radiological guidance. These patients undergo wire-guided BCS rather than intraoperative ultrasound–guided surgery, and were therefore not included in this study.

In the intraoperative US group, tumor margins were identified using a Clarius portable handheld ultrasound device. The superior, inferior, medial, and lateral borders of the tumor were marked on the skin using a surgical marker pen. In our surgical clinic, breast US is routinely employed during the preoperative evaluation. However, the intraoperative ultrasound device became available in the operating room later during the study period; thus, earlier patients underwent palpation-guided surgery, while later patients were operated on using intraoperative ultrasound guidance. All breast-conserving surgeries included in the study were performed by the same breast surgeon using standardized surgical techniques.

### Ethics approval and consent to participate

The study was conducted in strict accordance with the ethical principles outlined in the Declaration of Helsinki. It was approved by the Istanbul Medipol University Non-Interventional Clinical Research Ethics Committee on September 27, 2022, under approval number 822.

### Statistical analysis

Descriptive statistics were presented as frequency, percentage, mean, standard deviation, median, minimum, maximum, and 25th–75th percentiles (Q1–Q3). The assumption of normality was assessed using the Shapiro–Wilk test. Continuous variables exhibiting skewed distributions, such as resection volume, were primarily interpreted using median values and analyzed with non-parametric tests. For comparisons between two groups, the Independent Samples *t*-test was utilized for normally distributed numerical variables, while the Mann–Whitney *U* test was employed for non-normally distributed data.

Associations between categorical variables were analyzed using the Pearson chi-square test when less than 20% of the expected cell counts were below 5; otherwise, Fisher’s exact test was applied. In analyses involving more than two groups with statistically significant results, pairwise comparisons were performed using Bonferroni correction. Statistical analyses were conducted using Statistical Package for the Social Sciences (SPSS) version 23.0 (IBM Corp., Armonk, NY, USA). A *P* value < 0.05 was deemed statistically significant.

The primary outcomes of the study included resection volume, tumor diameter-to-resection volume ratio, and cavity re-excision. Other statistical comparisons were considered exploratory analyses and were interpreted accordingly.

Continuous variables were tested for normality using the Shapiro–Wilk test. Variables with skewed distributions, such as resection volume, were summarized using median and interquartile range (IQR) and analyzed using non-parametric tests. Multivariable logistic regression analysis was performed for cavity re-excision. Given that no final positive margins were observed in the study cohort, regression analysis for margin status was not conducted. Due to the retrospective design and limited number of events, adjusted analyses were interpreted cautiously, and results were presented as associations rather than causal effects.

## Results

The demographic, clinical, and pathological characteristics of the study population are summarized in Table 1. All tumors included in the study were clinically palpable breast lesions. Among the patients, 48.9% (*n* ═ 88) underwent surgery without the use of intraoperative US, while 51.1% (*n* ═ 92) underwent resection with intraoperative US guidance. The mean age of the patients was 54.82 years, with a median age of 53 years. Regarding menopausal status, 32.2% of patients were premenopausal and 67.8% were postmenopausal.

**Table 1:** Distribution of descriptive statistics for the study group

**Category**		***n***	**%**
Use of intraoperative ultrasound	No	88	48.9
	Yes	92	51.1
Menopausal status	Negative	58	32.2
	Positive	122	67.8
Histological subtype	Invasive Ductal	142	78.9
	Others	38	21.1
Grade	Grade 1	34	18.9
	Grade 2	108	60
	Grade 3	38	21.1
T	Tis	4	2.2
	T1 (not subclassified)	130	72.2
	T2	46	25.6
N	0	150	83.3
	1	30	16.7
Stage	Stage 0 (DCIS)	4	2.2
	Stage I	112	62.2
	Stage IIa or higher	64	35.6
Lymphovascular invasion	Negative	134	74.4
	Positive	46	25.6
Associated DCIS component	Negative	90	50
	Positive	90	50
Tumor necrosis	Negative	162	90
	Positive	18	10
ER	Negative	28	15.6
	Positive	152	84.4
PR	Negative	44	24.4
	Positive	136	75.6
HER2 receptor	Negative	162	91.1
	Positive	16	8.9
Tumor subtype	Luminal A	94	52.2
	Luminal B	44	24.4
	Triple negative	22	12.2
	HER2-enriched	16	8.8
Cavity re-excision	Not performed	156	86.7
	Performed	24	13.3
Axillary dissection	Not performed	156	86.7
	Performed	24	13.3
Tumor localization	Upper outer quadrant	128	71.1
	Others	52	28.9

Among the HER2-positive tumors (*n* ═ 16), 12 were hormone receptor-positive and categorized within the luminal B-like group. In contrast, 4 tumors were classified as ER-negative/PR-negative/HER2-positive, corresponding to the HER2-enriched subtype. Molecular subtype classification was exclusively applied to invasive breast carcinomas; patients with pure ductal carcinoma in situ (DCIS, stage 0) were excluded from this categorization. **Abbreviations:** DCIS: Ductal carcinoma in situ; ER: Estrogen receptor; HER2: Human epidermal growth factor receptor 2; N: Regional lymph node status; PR: Progesterone receptor; T: Primary tumor status; Tis: Tumor in situ.

Histopathological evaluation revealed invasive ductal carcinoma in 78.9% of patients, while 21.1% had other histopathological subtypes. According to T-stage distribution, 72.2% of patients were classified as T1, 25.6% as T2, and 2.2% as Tis. When subclassified, T1 tumors were categorized as T1a, T1b, and T1c according to AJCC 8th edition criteria. However, due to the retrospective nature of the study, T1 subclassification data were not consistently available for all patients and were therefore not included in the final analysis. Lymph node assessment revealed no metastasis in 83.3% of patients, whereas 16.7% had lymph node metastasis. Stage distribution analysis demonstrated Stage 0 (DCIS) in 2.2% of patients, 62% in stage I, and 35.6% in stage IIa or higher stages.

In terms of tumor grade, 18.9% of tumors were well differentiated, 60% were moderately differentiated, and 21.1% were poorly differentiated. Lymphovascular invasion was observed in 25.6% of patients, while 74.4% showed no evidence of lymphovascular invasion. Tumor necrosis was present in 10% of cases and absent in 90%. ER positivity was detected in 84.4% of patients, PR positivity in 75.6%, and HER2 overexpression in 8.9% of cases. Molecular subtype analysis among invasive breast carcinomas revealed luminal A tumors in 52.2% of patients, luminal B tumors in 24.4%, and triple-negative tumors in 12.2%. Among HER2-positive tumors (*n* ═ 16), 12 cases were hormone receptor-positive and classified as luminal B-like, whereas 4 cases were ER-negative/PR-negative/HER2-positive, corresponding to the HER2-enriched subtype.

Axillary dissection was performed in 13.3% of patients. Intraoperative cavity re-excision was performed in 13.3% of cases (*n* ═ 24). No patients required secondary re-operation, and all final pathological margins were negative. The presence of an associated DCIS component accompanying invasive carcinoma was observed in approximately half of the patients. Regarding tumor localization, 71.1% of tumors were located in the upper outer quadrant, while 28.9% were located in other quadrants.

The mean tumor diameter was 2.06 cm, with a median diameter of 2 cm. The mean resection volume was 128.36 cm^3^, with a median of 102.05 cm^3^. The mean closest surgical margin distance was 6.07 mm, with a median distance of 5 mm.

When patients were categorized into two groups based on the use of intraoperative US, no statistically significant differences were observed between the groups in terms of age, menopausal status, histopathological subtype, or tumor grade (Table 2). Similarly, the distribution of molecular subtypes, including luminal A, luminal B, and triple-negative tumors, was comparable between the two groups, with no statistically significant differences detected.

**Table 2:** Descriptive statistics for groups with and without intraoperative ultrasound utilization

**Intraoperative ultrasound use**		**No IOUS (*n* ═ 88)**		**IOUS (*n* ═ 92)**		
**Category**		***n***	**%**	**N**	**%**	***P***
Menopausal status	Negative	30	34.10%	28	30.40%	0.711
	Positive	58	65.90%	64	69.60%	
Histological subtype	Invasive ductal	72	81.80%	70	76.10%	0.505
	Others	16	18.20%	22	23.90%	
Grade	Grade 1	22	25.00%	12	13.00%	0.333
	Grade 2	50	56.80%	58	63.00%	
	Grade 3	16	18.20%	22	23.90%	
T	Tis	2	2.30%	2	2.20%	**0.009**
	T1	52	59.10%	78	84.80%	
	T2	34	38.60%	12	13%	
N	0	66	75.00%	84	91.30%	**0.038**
	1	22	25.00%	8	8.70%	
Stage	Stage 0 (DCIS)	2	2.30%	2	2.20%	**0.002**
	Stage I	40	45.50%	72	78.30%	
	Stage IIa or higher	46	52.30%	18	19.60%	
Lymphovascular invasion	Negative	54	61.40%	80	87.00%	**0.005**
	Positive	34	38.60%	12	13.00%	
Associated DCIS component	Negative	54	61.40%	36	39.10%	**0.035**
	Positive	34	38.60%	56	60.90%	
Tumor necrosis	Negative	86	97.70%	76	82.60%	**0.03**
	Positive	2	2.30%	16	17.40%	
ER	Negative	18	20.50%	10	10.90%	0.21
	Positive	70	79.50%	82	89.10%	
PR	Negative	26	29.50%	18	19.60%	0.27
	Positive	62	70.50%	74	80.40%	
HER2 receptor	Negative	84	95.50%	80	87.00%	0.26
	Positive	4	4.50%	12	13.00%	
Intraoperative cavity re-excision	Not performed	74	84.10%	82	89.10%	0.482
	Performed	14	15.90%	10	10.90%	
Final pathological margin status	Positive margin	0	0%	0	0%	_
Axillary dissection	Not performed	72	81.80%	84	91.30%	0.186
	Performed	16	18.20%	8	8.70%	
Tumor localization	Upper outer quadrant	72	81.80%	56	60.90%	**0.028**
	Others	16	18.20%	36	39.10%	

**Abbreviations:** DCIS: Ductal carcinoma in situ; ER: Estrogen receptor; HER2: Human epidermal growth factor receptor 2; IOUS: Intraoperative ultrasound; N: Regional lymph node status; PR: Progesterone receptor; T: Primary tumor status.

In the evaluation based on TNM staging, the non-US group exhibited more advanced T and N stages. Lymph node metastasis was more frequent in the group that did not utilize intraoperative ultrasound, and this difference was statistically significant (*P* ═ 0.038).

Regarding stage distribution, stage I disease was more prevalent in the intraoperative US group (78.3%), while stage IIa and higher stages were less frequent (*P* ═ 0.002). In the grouped stage analysis, lower-stage disease was also significantly more dominant in the US-guided group (*P* ═ 0.001).

Differences between groups were also observed in several pathological characteristics. Lymphovascular invasion, tumor necrosis, and the presence of an associated DCIS component exhibited statistically significant differences between the groups. These variables represent postoperative pathological findings that cannot be influenced by intraoperative ultrasound; thus, they should be interpreted as baseline imbalances between the study groups rather than effects of the intervention.

With respect to surgical characteristics, the rates of cavity re-excision and axillary dissection were similar between the two groups, with no statistically significant differences. However, tumor localization differed significantly; tumors located in the upper outer quadrant were less frequent in the intraoperative US group (60.9%) compared to the non-US group (81.8%), yielding a statistically significant difference (*P* ═ 0.028).

Comparisons of continuous variables between the intraoperative US and non-US groups are presented in Table 3. The mean tumor size in the US group was 14.59 ± 6.22 mm, with a median size of 15 mm. In contrast, the non-US group demonstrated larger tumors, with a mean size of 18.27 ± 8.21 mm and a median size of 20 mm. This difference was statistically significant (*P* ═ 0.019).

**Table 3:** Comparison of continuous variables between groups utilizing and not utilizing intraoperative ultrasound

**Continuous variables**	**Intraoperative ultrasound use**	***n***	**Mean** **± SD**	**Median (IQR)**	***P***
**Age**	Not performed	88	54.45 ± 11.22	52.5 (45–61)	0.756
	Performed	92	55.17 ± 10.68	54.5 (47–63)	
**Tumor size (mm)**	Not performed	88	18.27 ± 8.21	20 (47–63)	**0.019**
	Performed	92	14.59 ± 6.22	15 (10–20)	
**Tumor diameter (cm)**	Not performed	88	2.23 ± 0.77	2 (1.5–2.8)	**0.025**
	Performed	92	1.89 ± 0.64	2 (1.4–2.2)	
**Resection volume (cm^3^)**	Not performed	88	146.72 ± 95.33	130.91 (80.78–201.52)	**0.001**
	Performed	92	110.8 ± 187.65	64.65 (36.23–115.5)	
**Tumor diameter/resection volume**	Not performed	88	0.02 ± 0.02	0.01 (0.01–0.02)	**0.044**
	Performed	92	0.03 ± 0.02	0.02 (0.01–0.04)	
**Nearest surgical margin (mm)**	Not performed	88	6.52 ± 4.84	5 (3–8)	0.505
	Performed	92	5.63 ± 3.99	5 (3–7)	

All continuous variables are presented as mean ± SD and median (IQR) based on their distribution. **Abbreviations:** IQR: Interquartile range; SD: Standard deviation.

Similarly, when tumor diameter was evaluated, patients who underwent intraoperative US-guided surgery had significantly smaller tumor diameters compared to those in whom US was not used. In the US group, the mean tumor diameter was 1.89 ± 0.64 cm, with a median diameter of 2 cm, whereas the non-US group exhibited a larger mean tumor diameter of 2.23 ± 0.77 cm. This difference was also statistically significant (*P* ═ 0.025).

Resection volume was smaller in the intraoperative US group, with a mean value of 110.8 ± 187.65 cm^3^, compared to 146.72 ± 95.33 cm^3^ in the non-US group. The median resection volume was also lower in the US group (64.65 cm^3^), and this difference was statistically significant (*P* ═ 0.001), consistent with the non-parametric analysis applied due to the skewed distribution of this variable. Continuous variables with skewed distributions, including resection volume and tumor diameter-to-resection volume ratio, were summarized using median and IQR, in accordance with the statistical methods. Resection volume was significantly lower in the intraoperative ultrasound group, with a median of 64.65 cm^3^ (IQR: 36.23–115.5), compared to 130.91 cm^3^ (IQR: 80.78–201.52) in the palpation-guided group. Similarly, the tumor diameter-to-resection volume ratio was higher in the intraoperative ultrasound group (median 0.02, IQR: 0.01–0.04) compared to the palpation-guided group (median 0.01, IQR: 0.01–0.02).

The ratio of tumor diameter to resection volume was calculated as the pathological tumor diameter divided by the excised specimen volume. This ratio was significantly higher in the intraoperative US group, with a mean value of 0.03 ± 0.02, compared to 0.02 ± 0.02 in the non-US group (*P* ═ 0.044). A higher ratio indicates more precise tumor-targeted excision relative to the volume of removed breast tissue.

No statistically significant difference was observed between the intraoperative US and non-US groups regarding the closest surgical margin (*P* ═ 0.505).

A multivariable logistic regression analysis was performed to evaluate whether the use of intraoperative ultrasound was independently associated with cavity re-excision after adjustment for potential confounding variables, including tumor diameter, T stage, and lymphovascular invasion. In this adjusted model, intraoperative ultrasound use was not independently associated with the likelihood of cavity re-excision.

Our study also evaluated the variation in the tumor diameter-to-resection volume ratio and the need for cavity re-excision based on patients’ clinical and pathological characteristics.

Menopausal status, tumor grade, TNM stage, presence of an associated DCIS component accompanying invasive carcinoma, presence of necrosis, lymphovascular invasion status, molecular subtypes (luminal A, luminal B, and triple-negative), tumor localization, and axillary dissection status did not exhibit statistically significant effects on the tumor diameter-to-resection volume ratio. However, patients with invasive ductal carcinoma as the histopathological subtype and those who underwent cavity re-excision demonstrated a significantly higher tumor diameter-to-resection volume ratio compared to their counterparts, with *P* values of 0.042 and 0.047, respectively.

When comparing patients who underwent cavity re-excision to those who did not in terms of other clinical and pathological variables, a statistically significant difference was observed only concerning tumor localization.

No statistically significant differences were identified between patients with and without cavity re-excision regarding menopausal status, histopathological subtype, TNM staging, lymphovascular invasion, presence of associated DCIS component accompanying invasive carcinoma, presence of necrosis, axillary dissection status, or molecular subtypes (luminal A, luminal B, and triple-negative). In terms of tumor localization, tumors were situated in the upper outer quadrant in 66.7% of patients who did not require cavity re-excision, while 33.3% of these tumors were located in other quadrants. Notably, all tumors in patients who underwent cavity re-excision were located in the upper outer quadrant (*P* ═ 0.016).

Additionally, no statistically significant differences were observed when tumor localization was compared with resection volume and closest surgical margin. When patients with tumors located in the upper outer quadrant were contrasted with those in other quadrants, no significant differences emerged in terms of resection volume or closest surgical margin.

## Discussion

Breast cancer is the most common malignancy among women, with a lifetime incidence of approximately 12% [4]. Due to its high prevalence and the widespread implementation of screening programs, there has been a significant increase in early diagnosis, resulting in a growing number of patients diagnosed with early-stage breast cancer. Consequently, BCS is increasingly performed for both palpable and non-palpable small lesions. One of the major challenges in early-stage breast cancer surgery is achieving negative surgical margins. Frozen section analysis is commonly used intraoperatively to ensure margin negativity; however, errors associated with frozen section evaluation may necessitate secondary procedures such as cavity re-excision, thereby increasing healthcare costs. Moreover, reoperations may lead to delays in oncologic treatment, psychological distress, and suboptimal cosmetic outcomes.

The relatively lower proportion of grade 3 carcinomas in our cohort (21.1%) may be attributed to the study design. Since patients who received neoadjuvant therapy were excluded, biologically aggressive tumors—which are more frequently managed with preoperative systemic therapy—were underrepresented in this surgical population. Therefore, our findings reflect a selected early-stage surgical cohort rather than the overall breast cancer population.

Intraoperative US represents a low-cost, easily applicable, and widely accessible alternative method to address these challenges. In our study, patients who underwent resection under intraoperative US guidance had significantly smaller tumor diameters and resection volumes compared to those operated on without US guidance. Additionally, the tumor diameter-to-resection volume ratio was significantly higher in the intraoperative US group, indicating more precise tumor-targeted tissue excision while preserving surrounding healthy breast tissue. No significant differences were observed between the two groups regarding the closest surgical margin or the need for cavity re-excision. These findings suggest that the use of intraoperative US is associated with more precise tumor-targeted excision while maintaining adequate surgical margins.

It is crucial to note that the present study represents an initial exploration focused on clinically palpable breast tumors. However, existing literature indicates that intraoperative US is a safe and feasible technique for managing non-palpable breast lesions and for patients undergoing BCS after neoadjuvant therapy. In these contexts, intraoperative US may offer significant clinical advantages by enhancing lesion localization, improving surgical precision, and potentially decreasing re-excision rates. Therefore, although our findings pertain specifically to palpable tumors, the broader applicability of intraoperative US extends beyond this particular patient demographic [5].

A clear differentiation between intraoperative cavity re-excision, final pathological margin status, and secondary re-operation is essential for accurately interpreting surgical outcomes. In this study, intraoperative re-excision reflects real-time surgical decision-making, while final margin status serves as the definitive oncological endpoint. The absence of secondary re-operation in our cohort supports the adequacy of margin control achieved during the initial surgical procedure.

It is also important to recognize that several variables compared between the groups, such as lymphovascular invasion, tumor necrosis, and the presence of an associated DCIS component, represent postoperative pathological characteristics that cannot be altered by intraoperative US. Thus, these variables should be viewed as indicators of baseline differences between the groups rather than outcomes attributable to the intervention.

To address potential confounding factors associated with the retrospective study design, multivariable logistic regression analysis was conducted for the primary surgical outcomes. After adjusting for tumor diameter, T stage, and lymphovascular invasion, the use of intraoperative US was not independently associated with cavity re-excision. These findings suggest that the observed differences in surgical parameters may reflect baseline tumor characteristics rather than a direct causal effect of intraoperative US.

Given the limited number of events and the retrospective nature of the study, the adjusted analyses should be regarded as exploratory rather than confirmatory.

In a related study by Shah et al., involving 80 patients, it was found that specimen volume was smaller in those undergoing intraoperative US-guided surgery. The authors assessed the calculated resection ratio, defined as the ratio of excised specimen volume to optimal specimen volume, reporting significantly lower values in the US group compared to the palpation-guided group [6].

The COBALT study conducted by Krekel et al., one of the largest multicenter controlled trials evaluating the use of intraoperative US, demonstrated that excision volume and calculated resection ratios were significantly lower in US-guided resections than in palpation-guided procedures [5].

Similarly, Karanlık et al. reported lower resection volumes in a study of 164 patients undergoing intraoperative US-guided surgery compared to those operated on without US, despite similar tumor sizes [7].

In a 2023 study, Ferrucci et al. demonstrated that specimen volume was significantly smaller in intraoperative US-guided resections than in those performed using conventional palpation techniques. Additionally, the tumor volume-to-specimen volume ratio was lower in the US group, with both comparisons yielding statistically significant differences, indicating that intraoperative US effectively reduces unnecessary resections. The study also found superior margin negativity and lower re-excision rates in the US group [8].

Eggemann et al. compared margin negativity rates, intraoperative re-excision rates, and secondary surgical re-excision rates between US-guided and palpation-guided resections. Their findings indicated a significantly higher rate of negative margins in the US group, with both intraoperative and secondary re-excision rates being significantly lower in this cohort [9]. In contrast, our results did not reveal a significant difference between the two groups regarding the need for cavity re-excision or the proximity of the surgical margin.

Two studies in 2011 by Olsha et al. and Davis et al. also reported that intraoperative US was superior in achieving negative margins and reducing re-excision rates [10, 11]. With the routine use of frozen section analysis, negative margins can often be achieved intraoperatively, thereby minimizing the need for re-excision. In our study, cavity re-excision rates were low in both groups, and negative margins were attained in the majority of patients.

Almezaien et al. reported R0 resection rates of 97.5% in the intraoperative US group compared to 79% in the non-US group, with a statistically significant difference (*P* ═ 0.007) [12].

Two meta-analyses conducted by Pan et al. and Zhang et al. similarly demonstrated that intraoperative US-guided resections achieved higher rates of negative surgical margins compared to wire-guided and palpation-guided resections [13, 14].

Beyond the advantages of enhancing resection accuracy, achieving negative margins, and reducing re-excision rates, several studies have also evaluated the superiority of intraoperative US over frozen section analysis. Kumar et al. found that specimen-oriented US assessment achieved 100% accuracy in ensuring negative margins, while frozen section analysis demonstrated an accuracy of 90.82% [15]. In a 2025 study, Ferrucci et al. reported that intraoperative US-guided surgery may enhance surgical precision and cosmetic outcomes, further supporting the expanding role of this technique in breast surgery [16].

The findings of this study should be interpreted as associations rather than causal effects due to the retrospective design and potential confounding factors.

### Limitations

This study has several limitations. First, its retrospective design may introduce selection bias and confounding factors. Additionally, there is an imbalance in tumor size between the two study groups, with patients in the intraoperative ultrasound group having significantly smaller tumors compared to those in the palpation-guided group. Since tumor size directly influences the extent of surgical excision, this discrepancy may have contributed to the observed differences in resection volume. Although tumor diameter was included as a covariate in the multivariable regression analysis, this imbalance must be considered when interpreting the findings. The introduction of intraoperative ultrasound occurred later in the study period, thus the comparison between the two groups reflects a change in surgical practice over time rather than a randomized patient allocation. While all procedures were performed by the same surgeon, the temporal introduction of intraoperative ultrasound may indicate a shift in surgical practice. Consequently, the findings should be interpreted as associations rather than causal relationships.

Another potential limitation is the learning curve associated with intraoperative ultrasound guidance. Although all procedures were conducted by the same breast surgeon, who routinely employs breast US in preoperative evaluations, the later implementation of intraoperative ultrasound may still involve a learning curve that cannot be entirely dismissed.

Moreover, the study exclusively included patients with clinically palpable breast tumors, limiting the generalizability of the findings to those with non-palpable breast lesions, which are typically managed through wire localization or other image-guided techniques.

Additionally, multiple secondary comparisons were performed, necessitating caution in interpreting these results as exploratory findings.

The delayed introduction of intraoperative ultrasound may also have introduced calendar-time confounding that could not be fully adjusted for in the analysis.

Furthermore, the limited number of events restricted the ability to conduct robust multivariable analyses with reliable effect estimates and confidence intervals.

## Conclusion

With the rising incidence of breast cancer, increased implementation of screening programs, and heightened public awareness, the proportion of patients diagnosed with early-stage breast cancer continues to grow. This trend is accompanied by a higher frequency of non-palpable or small lesions, complicating the determination of appropriate surgical margins and potentially leading to unnecessary resections that significantly reduce breast volume.

Intraoperative US is a cost-effective and accessible method that may enhance surgical precision in BCS. Our findings indicate that its use is associated with more precise tumor-targeted excision and better preservation of surrounding breast tissue. Although numerous studies have highlighted the benefits of intraoperative ultrasound in achieving negative surgical margins and reducing re-excision rates, evidence regarding its role in preventing unnecessary tissue excision and improving cosmetic outcomes remains limited.

In our study, the tumor diameter-to-resection volume ratio was significantly higher in patients undergoing BCS with intraoperative US guidance, suggesting more precise tumor-targeted excision and better preservation of surrounding healthy breast tissue.

We advocate for future multicenter studies involving larger patient populations to provide more robust evidence clarifying the clinical benefits of intraoperative US in BCS.

7

**AI statement:** During the preparation of this manuscript, ChatGPT (OpenAI) was used solely for language editing and improving the readability of the text. The authors reviewed and edited the content as necessary and take full responsibility for the final manuscript.

## Data Availability

The relevant datasets are available upon reasonable request from the corresponding author.
